# aRrayLasso: a network-based approach to microarray interconversion

**DOI:** 10.1093/bioinformatics/btv469

**Published:** 2015-08-17

**Authors:** Adam S. Brown, Chirag J. Patel

**Affiliations:** Department of Biomedical Informatics, Harvard Medical School, Boston, MA 02115

## Abstract

**Summary:** Robust conversion between microarray platforms is needed to leverage the wide variety of microarray expression studies that have been conducted to date. Currently available conversion methods rely on manufacturer annotations, which are often incomplete, or on direct alignment of probes from different platforms, which often fail to yield acceptable genewise correlation. Here, we describe aRrayLasso, which uses the Lasso-penalized generalized linear model to model the relationships between individual probes in different probe sets. We have implemented aRrayLasso in a set of five open-source R functions that allow the user to acquire data from public sources such as Gene Expression Omnibus, train a set of Lasso models on that data and directly map one microarray platform to another. aRrayLasso significantly predicts expression levels with similar fidelity to technical replicates of the same RNA pool, demonstrating its utility in the integration of datasets from different platforms.

**Availability and implementation:** All functions are available, along with descriptions, at https://github.com/adam-sam-brown/aRrayLasso.

**Contact:** chirag_patel@hms.harvard.edu

**Supplementary information:** Supplementary data are available at *Bioinformatics* online.

## 1 Introduction

A pressing issue in translational biology is the ability to reference and utilize historical microarray datasets for large-scale discovery programs (Tsiliki *et* *al.*, 2014). The appeal of using historical datasets includes capturing previous investment to construct larger cohorts. Despite interest in both industry and academia (Tsiliki *et* *al.*, 2014; Yengi, 2005), few groups have attempted to tackle the problem of platform integration. Current approaches primarily rely upon passing different microarray platforms through a common identifier system, such as EntrezGene IDs, using specially designed packages (Alibes *et al.*, 2007; Mohammad *et* *al.*, 2012) or online tools (Huang *et* *al.*, 2009). While these systems work well in cases where manufacturers have maintained annotations of their microarray databases, ID-based conversion methods fail for deprecated and undermaintained microarray platforms. Another approach to convert between platforms is sequenced-based, wherein each sequence tag is aligned to the genome or transcriptome and annotated (Fumagalli *et* *al.*, 2014; Liu *et* *al.*, 2007). Unfortunately, it is often the case that *de novo* annotations do not capture the complexity of the transcriptome (e.g. for genes with alternative splice variants Gambino *et* *al.*, 2015).

To address the shortcomings of both annotation- and sequence-based conversion methods, we have developed aRrayLasso, a Lasso-regression based network model. Our method directly predicts the probe expression levels of the target platform. To demonstrate the accuracy of our method, we show that predictions made using aRrayLasso are of similar accuracy to technical replicates from the 6 same mRNA pool. Our methodology allows users to utilize currently available methodologies for integrating cross-experiment microarray datasets (Tsiliki *et* *al.*, 2014) and allow for the construction of large-cohort retrospective studies.

## 2 Methods

**Fig. 1.**
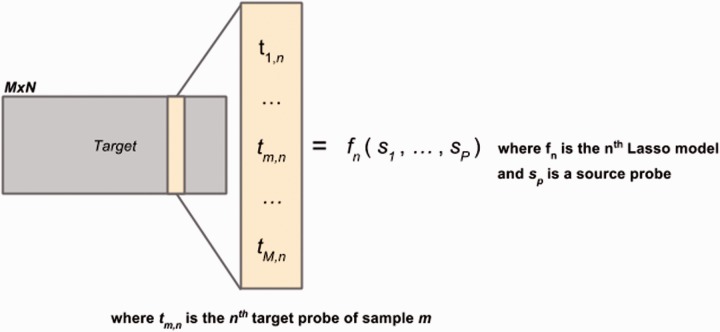
Schematic of the aRrayLasso algorithm. aRrayLasso takes in an *MxN* target matrix containing *M* samples and *N* probes. A Lasso model, *f_n_,* is then constructed for each target probe using all probes in the *MxP* source matrix (*M* samples, *P* probes)

To convert from a source to a target microarray platform, we chose to model each individual sequence tag in the target platform as a linear combination of all sequence tags from the source platform (see Fig. 1 and Supplementary Methods). Because microarrays have greater than 10 000 individual probes, we chose to use the Lasso algorithm for generalized linear regression (Friedman *et* *al.*, 2010). The Lasso algorithm allows the resulting linear model to be ‘sparse’ in that only the most relevant and robust (by cross-validation) predictors are assigned non-zero values. This optimization allows the model to outperform similar models that require all predictors to be assigned non-zero coefficients (Tibshirani *et* *al.*, 2010). Lasso is implemented in the R package ‘glmnet,’ allowing for ease of use (Friedman *et* *al.*, 2010).

We first generate a list of lasso models for each sequence tag in the target microarray platform. Our implementation can take as input a variety of data formats, including expression matrices, R expressionSet objects and Gene Expression Omnibus accession numbers (Edgar *et* *al.*, 2002). Once the full list of models has been computed, we provide functions that allow either the straightforward prediction of sequence tag values or the validation of the model list by calculation of Pearson product-moment correlation coefficients.

To demonstrate the utility of our methodology, we utilized three datasets: (i) GSE6313, containing C57/B6 adult mouse retina cDNA profiles (Liu *et* *al.*, 2007), (ii) GSE7785, containing PANC-1 derived cDNA profiles (Tan *et* *al.*, 2003) and (iii) GSE4854, containing mouse cortex expression profiles (Kuo *et* *al.*, 2006). Each dataset is composed of multiple technical replicates for several distinct microarray platforms (see Supplementary Table S1). For both datasets, we used aRrayLasso to first train models to intraconvert between each individual platform and then predicted intraconversions between each pair of platforms for all technical replicates. To assess the accuracy of our conversions, we calculated the average Pearson’s *r* between the predicted values and actual experimental values for each platform and replicate. We also calculated the average inter-replicate Pearson’s *r* for each platform (see Supplementary Table S2).

## 3 Results

To explore the performance of aRrayLasso, we began by comparing our method’s ability to predict expression to the biological variation between replicates on the same platform. We assessed the degree to which aRrayLasso could accurately predict platform interconversions in three datasets, representative of different experimental systems, organisms and platforms. For the five platforms tested, aRrayLasso predictions are within the technical variation of each microarray platform when compared with technical replicates from the same cDNA pool, even when subjected to multiple sequential conversions (Supplementary Table S2). In addition, once built, aRrayLasso models can be used between experimental conditions: using the models built on GSE6313, we predicted expression levels in GSE4854 with no significant loss of signal (Pearsons product-moment correlation, *P* < 0.38). While the results presented here do not guarantee similar results for all training and testing datasets, these analyses serve as a promising proof of concept. Furthermore, our success with a relatively small dataset suggests that aRrayLasso may reach even higher levels of performance as the size of the datasets involved increases.

## 4 Discussion

**Implementation**: In this investigation, we propose a data-driven method for integrating across high-throughput genomic measurement modalities that avoids the use of annotation- or sequence alignment-based tools. We have implemented a Lasso regression-based modeling approach to model the expression level of each sequence tag in a target microarray as a linear combination of all sequence tags in a source microarray. Our implementation represents a straightforward, easy-to-use and open-source methodology for conversion between microarray platforms.

**Limitations**: One drawback of our method is the need for extant or newly generated matched samples in the source and target platforms. In our experience, however, there are a large number of datasets available that have matched samples with replicates for a number of popular microarray platforms. A second limitation to our method is in conversion which lack overlap in gene coverage. In these cases, as with currently available methodologies, our method will fail to provide meaningful conversions. Lastly, while we have shown in one case that interexperiment conversions are feasible, we caution that systematic technical error in a single experiment may lead to the creation of a biased model. In general, however, when coupled with one of several cross-experiment dataset integration tools, aRrayLasso will enable mining of the remarkable and untapped historical pool of microarray datasets for large-scale metastudies for well-powered discovery.

## Supplementary Material

Supplementary Data
