# Supplementary material for: aRrayLasso: a network-based approach to microarray interconversion
Source: Bioinformatics. 2015 Aug 17;31(23):3859–61. doi: 10.1093/bioinformatics/btv469 (PMC4653393; doi:10.1093/bioinformatics/btv469)
Supplement: Supplementary Data [file supp_31_23_3859__index.html]

aRrayLasso: a network-based approach to microarray interconversion — aRrayLasso: a network-based approach to microarray interconversion — aRrayLasso: a network-based approach to microarray interconversion — Supplementary Data 

# aRrayLasso: a network-based approach to microarray interconversion

## Supplementary Data

files

- Supplementary Data - doc file
