# Supplementary material for: aRrayLasso: a network-based approach to microarray interconversion
Source: Bioinformatics. 2015 Aug 17;31(23):3859–61. doi: 10.1093/bioinformatics/btv469 (PMC4653393; doi:10.1093/bioinformatics/btv469)
Supplement: Supplementary Data [file supp_btv469_Supplement.doc]

**Supplementary Table 1.** Training and testing sets for all reported aRrayLasso results.

| GEO Accession | Platform | Training Set | Test Set |
| --- | --- | --- | --- |
| GSE6313 | [MG_U74Av2] Affymetrix Murine Genome U74A Version 2 Array | GSM108158  GSM108159  GSM108160  GSM108161  GSM108162 | GSM146212  GSM146213  GSM146214  GSM146215  GSM146216 |
|  | ABI Mouse Genome Survey Microarray | GSM108124  GSM108125  GSM108128  GSM108129  GSM108131 | GSM146230  GSM146231  GSM146232  GSM146233  GSM146234 |
|  | GE-Amersham-CodeLink-10K | GSM108941  GSM108942  GSM108944  GSM108945 | GSM146217  GSM146218  GSM146219  GSM146220  GSM146221 |
| GSE7785 | GE Healthcare/Amersham Biosciences CodeLink™ UniSet Human I Bioarray | GSM188556  GSM188570  GSM188571  GSM188572  GSM188573 | GSM188574  GSM188575  GSM188576  GSM188577 |
|  | [HG_U95Av2] Affymetrix Human Genome U95 Version 2 Array | GSM198009  GSM198010  GSM198011  GSM198012  GSM198013 | GSM198014  GSM198016  GSM198017  GSM198018 |
| GSE4854 | [MG_U74Av2] Affymetrix Murine Genome U74A Version 2 Array | Models generated using GSE6313 training set | GSM108110  GSM108116  GSM108118  GSM108120  GSM108122 |
|  | ABI Mouse Genome Survey Microarray | Models generated using GSE6313 training set | GSM108163  GSM108164  GSM108165  GSM108166  GSM108167 |

**Supplementary Table 2.** Average Pearson's r for all reported aRrayLasso Results

| GEO Accession | Conversion | Average Pearson's r | Average inter-replicate Pearson's r | Fisher transformation z-score p value* |
| --- | --- | --- | --- | --- |
| GSE6313 | ABI to Affymetrix | 0.4678682 | 0.3873569 | 0.4602 (N.S.) |
|  | ABI to Amersham | 0.2195729 | 0.1980275 | 0.492 (N.S.) |
|  | Affymetrix to ABI | 0.141759 | 0.09751847 | 0.484 (N.S.) |
|  | Affymetrix to Amersham | 0.2268909 | 0.1980275 | 0.488 (N.S.) |
|  | Amersham to ABI | 0.13333801 | 0.09751847 | 0.484 (N.S.) |
|  | Amersham to Affymetrix | 0.4567984 | 0.3873569 | 0.4681 (N.S.) |
|  | ABI to Affymetrix to Amersham to ABI | 0.4310321 | 0.09751847 | 0.3594 (N.S.) |
| GSE7785 | Affymetrix to Amersham | 0.091541 | 0.0655411157 | 0.492 (N.S.) |
|  | Amersham to Affymetrix | 0.0482165103 | 0.1465847167 | 0.4721 (N.S.) |

*The Fisher transformation provides a method of converting Pearson's r values to z scores, which in turn allows for the straightforward calculation of a p-value for whether two Pearson's r values are significantly different.

**Supplementary Methods.** Detailed description of algorithm used for aRrayLasso model construction.

1. Select a source microarray platform (the platform to be converted **from**) and a target microarray platform (the platform to be converted **to**)
2. Identify or create a dataset where a number of samples (at least 5) have been profiled using both the target and source microarray platforms.
3. For each sequence tag (probe) in the target platform, do the following:
   1. Construct a generalized linear regression model (Lasso) with the target probe being the dependent variable and all of the probes in the source platform being the explanatory variables of the form

| $t_{n}=\epsilon+\sum_{i=1}^{M} \left(s_{i}*c_{i}\right)$ | (Eqn 1) |
| --- | --- |

Where *t_n_* is the n^th^ sequence tag of the target platform (of *N* total), ε is a constant, *s_i_* is the i^th^ sequence tag of the source platform, *c_i_* is the i^th^ linear regression constant, and *M* is the total number sequence tags in the source platform.

- 1. Store the the model in a named list for later accessing.
